# The Intersection between Frailty, Diabetes, and Hypertension: The Critical Role of Community Geriatricians and Pharmacists in Deprescribing

**DOI:** 10.3390/jpm14090924

**Published:** 2024-08-30

**Authors:** Daniel Dinarvand, Johann Panthakey, Amirmohammad Heidari, Ahmed Hassan, Mohamed H. Ahmed

**Affiliations:** 1 Department of Medicine, Ashford and St. Peter’s Hospital NHS Foundation Trust, Surrey KT16 0PZ, UK; daniel.dinarvand@nhs.net; 2 Department of Medicine, Royal Surrey County Hospital NHS Foundation Trust, Guildford GU2 7XX, UK; johann.panthakey@nhs.net; 3 Department of Trauma and Orthopaedics, Liverpool University Hospitals NHS Foundation Trust, Liverpool L7 8YE, UK; 4 Faculty of Medicine, Alexandria University, Alexandria 21321, Egypt; ahmed.mohamed2133@alexmed.edu.eg; 5 Department of Medicine and HIV Metabolic Clinic, Milton Keynes University Hospital NHS Foundation Trust, Eaglestone, Milton Keynes MK6 5LD, UK; 6 Department of Geriatric Medicine, Milton Keynes University Hospital NHS Foundation Trust, Eaglestone, Milton Keynes MK6 5LD, UK; 7 Honorary Senior Lecturer of the Faculty of Medicine and Health Sciences, University of Buckingham, Buckingham MK18 1EG, UK

**Keywords:** frailty, diabetes, hypertension, community geriatrician

## Abstract

**Background**: Frailty is a clinical syndrome prevalent among the elderly, characterised by a decline in physiological reserves and increased susceptibility to stressors, resulting in higher morbidity and mortality. Diabetes and hypertension are common in frail older individuals, often leading to polypharmacy. In this narrative review, we aimed to evaluate the relationship between frailty, diabetes, and hypertension and to identify effective management strategies and future research directions. **Methods**: This narrative review was conducted using the Scopus, Medline, PubMed, Cochrane Library, and Google Scholar databases. **Results:** Frailty significantly impacts the management and prognosis of diabetes and hypertension, which, in turn, affects the progression of frailty. Managing these conditions often involves multiple drugs to achieve strict glycaemic control and blood pressure targets, leading to polypharmacy and associated morbidities, including orthostatic hypotension, falls, fractures, hypoglycaemia, and reduced medication adherence. Identifying frailty and implementing strategies like deprescribing can mitigate the adverse effects of polypharmacy and improve outcomes and quality of life. Despite the availability of effective tools for identifying frailty, many frail individuals continue to be exposed to complex treatment regimens for diabetes and hypertension, leading to increased hospital admissions, morbidity, and mortality. **Conclusions:** Managing diabetes and hypertension in the frail ageing population requires a multidisciplinary approach involving hospital and community geriatricians and pharmacists. This is important due to the lack of sufficient clinical trials dedicated to diabetes and hypertension in the context of frailty. Future large population studies are needed to assess the best approaches for managing diabetes and hypertension in frail individuals.

## 1. Introduction

Frailty is characterised by a deterioration in the function of the body’s organ systems and a decline in physiological reserves, leading to heightened susceptibility to external stressors and a reduced ability to recover to baseline, ultimately resulting in negative health consequences [1,2]. The presence of frailty in the elderly population can manifest as a decline in physical strength, endurance, and overall physiological function, and also difficulties in managing daily tasks, responding to sudden stresses, or recovering from morbidity [3,4]. It is multifactorial, involving multiple physiological systems, and is linked to significant morbidity, including an increased risk of falls, disability, hospitalisation, reduced quality of life, morbidity, and a huge burden on health systems across the globe. [5,6]. The condition affects a substantial proportion of the elderly population, with prevalence rates varying widely depending on the definition and population studied. Approximately 10–15% of those aged 65 and over, and up to 25–50% of those aged 85 and above, are frail to varying extents and this will represent considerable challenges for healthcare systems and caregivers globally due to the increased need for medical and supportive care [5,6,7]. Social frailty refers to a decline in social relationships and support, which can exacerbate the physical and cognitive aspects of frailty [8]. Identifying frailty at an early stage increases the potential for therapeutic opportunities. There is evidence to show that interventions such as physical activity, nutritional support, and cognitive training can improve outcomes and quality of life, potentially postponing or reversing the progression of frailty and its impact on the likelihood of negative health outcomes [9,10,11]. 

To effectively manage frailty, a multidisciplinary approach is needed, involving various healthcare practitioners, including geriatricians, primary care physicians, nurses, physiotherapists, and pharmacists. A collaborative care model enhances the ability to address the complex needs of frail patients, ensuring comprehensive care and promoting better health outcomes [12]. 

Frailty is commonly observed in older adults and is closely interconnected with diabetes and hypertension, with these conditions often exacerbating the impact of each other. Studies have shown that diabetes exacerbates frailty by increasing the risk of muscle weakness, poor mobility, and overall physical decline [13,14]. Similarly, hypertension, which often coexists with diabetes, further contributes to frailty through its impact on cardiovascular health, leading to a higher risk of adverse outcomes such as falls, disability, and mortality [15]. Addressing these conditions holistically is crucial, as effective management of diabetes and hypertension can mitigate the progression of frailty, thereby improving quality of life and reducing healthcare burdens [16].

Importantly, the development of hypertension may be an early feature of insulin resistance and hence this may explain in part the common association between diabetes and hypertension [17,18]. A global study conducted between 1990 and 2019 reported a substantial rise in the global burden of type 2 diabetes. It alluded that while life expectancy for individuals with type 2 diabetes has increased, this has been accompanied by significant increases in morbidity, mortality, and years lived with disability. The total number of DALYs due to type 2 diabetes was reported as 70.88 million in 2019, reflecting the growing burden of the disease and demonstrating the continued increase in prevalence, mortality, and disability among those with type 2 diabetes, despite advances in management and treatment [19]. Furthermore, the prevalence of diabetes in older adults is estimated to be 33% and this number is expected to increase significantly in the coming years [20].

Diabetes management in the elderly population is a complex issue due to comorbidities, recurrent hospital admissions, exaggerated consequences of adverse effects from treatment, the tendency for hypoglycaemia, falls, fractures, and shortened life expectancy. Overtreatment of diabetes in the elderly population is common as when patients are in good health or admitted to hospitals, their diabetes treatment is intensified to achieve glycaemic, lipid, and blood pressure targets. Therefore, assessment of frailty and appropriate deprescribing should be part of the assessment of all frail patients living with diabetes [21].

Hypertension and diabetes are both associated with an increased risk of the development of frailty [22,23]. A high prevalence of hypertension has also been reported in elderly populations. For instance, the global prevalence of hypertension is shown to increase with age, from 27% in patients aged younger than 60 years to 74% in those aged older than 80 years. The Framingham Heart Study showed that more than 90% of the normotensive participants between 55 and 65 years developed hypertension [24,25]. 

Research and clinical trials that studied the management of hypertension in the elderly population excluded frail individuals. There is a lack of guidance for recommended blood pressure targets in severely frail patients. Bogaerts et al. provided a systematic overview of the thresholds and targets for blood pressure management in older people amongst 42 blood pressure guidelines worldwide (34 of which made recommendations for blood pressure management in ageing and/or frailty). Their conclusion is that 20 guidelines, including the National Institute for Health and Care Excellence (NICE) guidelines, recommend systolic blood pressure targets < 150 mmHg for the elderly [22].

Recruiting frail individuals for clinical research is challenging due to their vulnerability and comorbidities. Frail individuals often have limited mobility, cognitive impairments, and a higher risk of adverse events, which can deter participation. Ethical concerns and the need for specialised care during studies further complicate recruitment efforts. Strategies to enhance recruitment include tailored communication, providing transportation, and ensuring caregiver support [26]. There is a need for evidence-based medicine in the management of frail old individuals with diabetes and hypertension. In this narrative review, we aimed to provide the most recent evidence of the management of diabetes and hypertension in the frail elderly population. 

## 2. Methods

**Figure 1:**
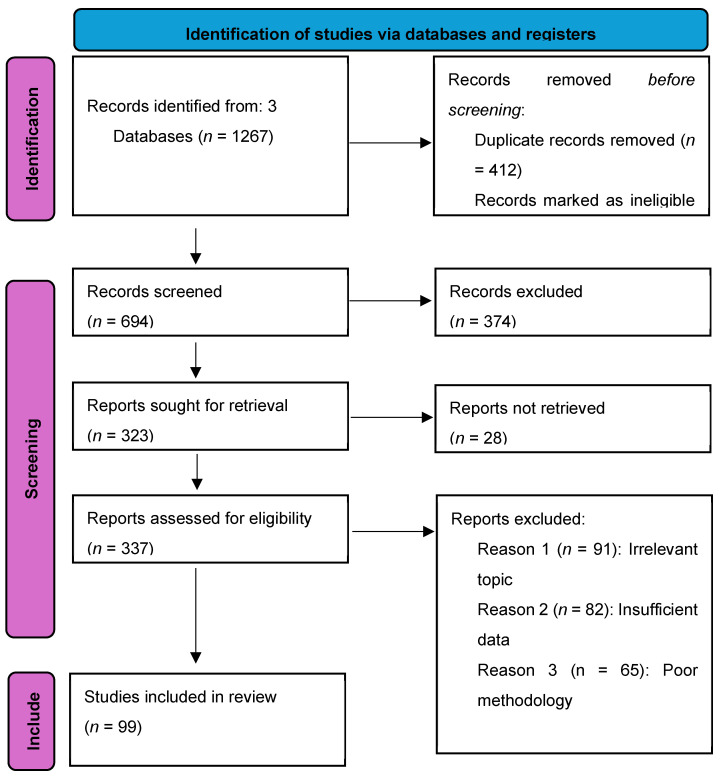
PRISMA flow diagram illustrating the systematic search and selection process undertaken for this review (https://www.prisma-statement.org/prisma-2020-flow-diagram, accessed on 20 August 2024).

In this narrative review, a review of the literature was conducted and the following electronic databases were searched: Scopus, PubMed, Medline, Cochrane, and Google Scholar. The literature search process used a combination of keywords to ensure comprehensive coverage of relevant studies. The keywords used in the database search were as follows: (“frailty” OR “frail”) (“diabetes” OR “type 1 diabetes” OR “type 2 diabetes” OR “hyperglycaemia”) (“hypertension” OR “high blood pressure” OR “hypertensive” OR “blood pressure”) (“older adults” OR “elderly” OR “geriatrics” OR “aging population” OR “seniors”) (“polypharmacy”) (“deprescribing”). After identifying the locations of the required articles, some of them were retrieved electronically and some were retrieved manually by searching libraries in our hospitals. Studies were included if they met the following criteria: focused on frailty, diabetes, and hypertension, and involved older adults (aged 65 and above). We excluded studies that looked at diabetes and hypertension in those less than 65 years old and were not written in English. Data from the included studies were extracted and synthesised narratively to provide an overview of current research on the intersection of frailty, diabetes, and hypertension in older adults. Figure 1 shows how the literature search was conducted using the PRISMA flow diagram and the number of studies included in this review article.

## 3. Clinical Tools for Assessing Frailty

### 3.1. Fried Frailty Phenotype

The Fried Frailty Phenotype is a widely used and well-validated tool for assessing frailty. It classifies an individual as frail in the presence of at least three of the following five criteria: involuntary weight loss (>4.5 kg), weakness or inadequate handgrip strength, self-reported fatigue, reduced walking speed, and low levels of physical activity [6]. The Fried Frailty Phenotype has undergone extensive evaluation and validation. Research has shown that it is useful in identifying frailty and predicting negative health outcomes, such as disability, hospitalisation, and mortality. It has been shown to be applicable across different demographic groups and ethnicities, providing consistent results in identifying frail older individuals and directing interventions to improve their health outcomes [6,27,28,29].

### 3.2. Clinical Frailty Scale

The Clinical Frailty Scale (CFS), described by Rockwood et al. [30], is another important tool for assessing frailty. In contrast to more complex and time-consuming frailty models, the CFS approaches the assessment of frailty in a simpler manner, making it especially useful in clinical settings. Emerging from the work of the Canadian Study of Health and Ageing (CSHA), the Clinical Frailty Scale assesses frailty subjectively. A healthcare professional can use their clinical judgement to grade a patient’s frailty using a nine-point scale. The CFS is simple and easy to use, allowing healthcare professionals to assess frailty quickly in various settings, including primary and secondary care (Table 1). Higher CFS scores are strongly linked to an increased risk of rehospitalisation, institutionalisation, and mortality [31]. Hubbard et al. [32] found that the CFS was effective in predicting in-hospital mortality and length of stay, providing a useful means of stratifying patients with risk of poor outcomes and helping to appropriately allocate healthcare resources and plan individualised care. A disadvantage of the CFS is its subjectivity, meaning the score given to an individual may vary depending on the clinical judgement of the practitioner.

### 3.3. Edmonton Frail Scale

The Edmonton Frail Scale (EFS) is a comprehensive tool used to assess frailty in elderly patients and it evaluates multiple domains (Table 2). The total score ranges from 0 to 17, with higher scores indicating greater frailty. The scale helps in identifying vulnerable patients who may benefit from targeted interventions to improve their health and quality of life [33].

### 3.4. Comprehensive Geriatric Assessment

The Comprehensive Geriatric Assessment (CGA) is a multimodal, interdisciplinary diagnostic method that assesses comorbidities, psychological state, and functional skills in the elderly. This strategy is effective in meeting the complex health demands of the elderly [34,35]. The CGA assesses multiple domains: medical, cognitive, emotional, functional, social, and environmental [36]. Evaluation focuses on identifying acute and chronic disorders, as well as reviewing medications to prevent polypharmacy. Cognitive assessments measure memory, attention, and other cognitive skills to detect disorders such as dementia or delirium. Emotional assessments look for sadness, anxiety, and other mental health problems. The functional evaluation determines an individual’s capacity to execute activities of daily living (ADLs) and instrumental activities of daily living (IADLs). Social assessment investigates the patient’s support network, living situation, and financial resources. Environmental assessments measure the safety and accessibility of the home environment [37,38]. Utilisation of the CGA can improve outcomes in the elderly by increasing functional status, lowering hospitalisations, and improving quality of life [39].

Research has shown the CGA’s utility in improving health outcomes. Stuck et al. [38] conducted a major meta-analysis of CGA therapies, demonstrating that CGA dramatically increased the likelihood of patients living at home, decreased the risk of functional deterioration, and reduced mortality rates. This study highlighted CGA’s comprehensive approach to providing holistic, patient-centred care for older persons. Rubenstein et al. [37] conducted one of the first randomised controlled trials of CGA, showing that patients who underwent CGA had better functional results and were less likely to be institutionalised than those who received standard treatment. Similarly, Ellis et al. [39] found that inpatient CGA improved survival and increased the likelihood of staying at home following discharge. The CGA is also useful in outpatient and community settings. Landefeld et al. [40] found that older persons who received CGA in an outpatient setting had fewer hospital admissions and emergency department visits and reported a higher quality of life. The study emphasised the importance of CGA in managing chronic diseases and preventing acute presentations to hospitals, reducing the load on healthcare systems. Theou et al. [41] showed that CGA in nursing homes and other long-term care facilities can increase functional abilities, reduce hospitalisations, and improve overall well-being. This study showed that CGA is adaptable and useful in various care settings, reinforcing its importance in the care of the elderly. 

**Figure 2:**
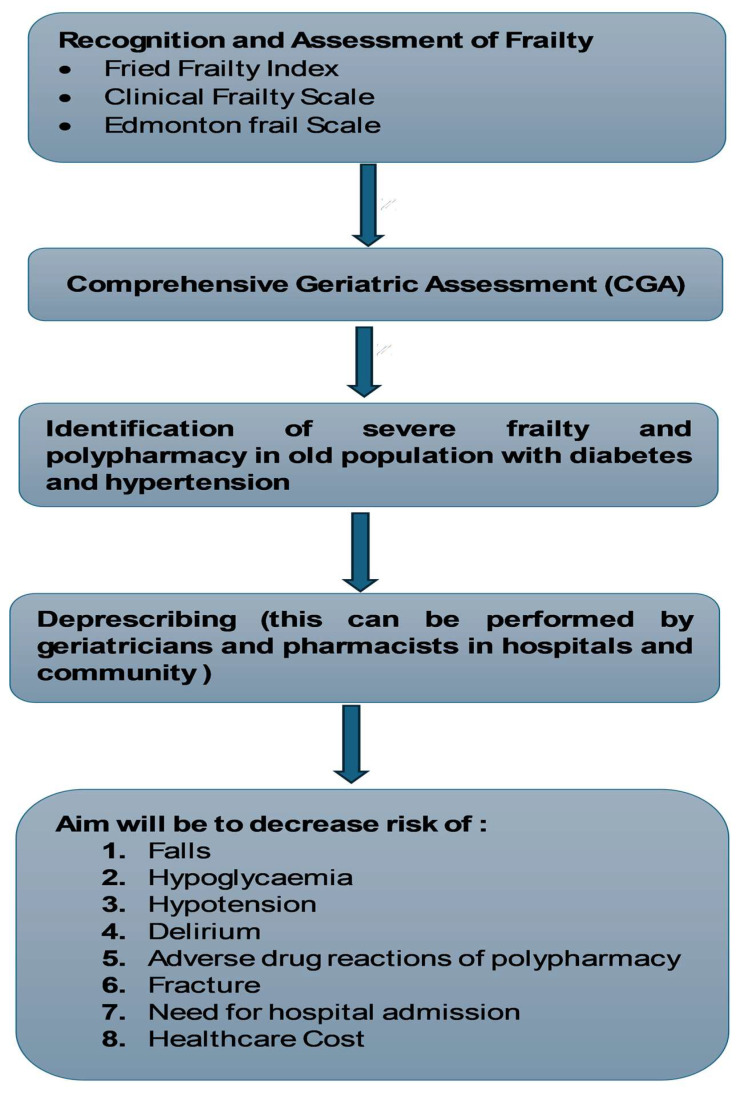
Benefits of using frailty scales and comprehensive geriatric assessment to identify opportunities for deprescribing in severely frail individuals living with diabetes and hypertension.

The interdisciplinary approach of the CGA, which includes primary care physicians, geriatricians, nurses, social workers, physical therapists, occupational therapists, and other healthcare specialists, ensures a comprehensive and coordinated approach to care. This collaboration is vital for meeting the diverse requirements of the elderly and developing personalised and holistic care plans. Importantly, CGA can be completed in hospitals and the community, thus assisting in the decision-making for safe and confident deprescribing of antihypertensive and antidiabetic medications. The benefits of such practices are highlighted in Figure 2.

## 4. Diabetes and Frailty

The relationship between diabetes and frailty is an important area of research given the growing prevalence of both conditions among older adults [42,43]. Considering this relationship is crucial for developing targeted interventions and improving health outcomes in this population. 

Frailty has been identified as a substantial risk factor associated with diabetes. The mechanisms underlying this association are multifaceted, encompassing inflammatory, vascular, and metabolic pathways. Chronic hyperglycaemia is strongly linked to the development and progression of frailty in older adults through multiple interconnected mechanisms. One of the key pathways is chronic inflammation. Persistent hyperglycaemia is associated with elevated levels of pro-inflammatory cytokines, such as interleukin-6 (IL-6) and tumour necrosis factor-alpha (TNF-α), which drive systemic inflammation. This low-grade chronic inflammation contributes to the breakdown of muscle tissue (sarcopenia), reduced physical function, and overall diminished physiological resilience, all of which are hallmarks of frailty [44,45]. 

In addition to inflammation, hyperglycaemia also induces oxidative stress by increasing the production of reactive oxygen species (ROS). Oxidative stress damages cellular structures, including proteins, lipids, and DNA, and this damage accelerates the ageing process, leading to declines in muscle strength, mobility, and cognitive function, which are key features of frailty [45]. The accumulation of advanced glycation end products (AGEs) due to prolonged hyperglycaemia also exacerbates oxidative stress, contributing to muscle weakness and frailty by impairing muscle protein function and increasing stiffness in tissues [44]. 

Endothelial dysfunction is another critical consequence of hyperglycaemia. The vascular damage caused by prolonged elevated blood glucose levels reduces blood flow and impairs oxygen delivery to vital organs, including the muscles and brain. This dysfunction increases the risk of cardiovascular diseases and stroke, which further contributes to the development of frailty by impairing mobility and physical independence [45]. The accumulation of advanced glycation end products (AGEs) due to chronic hyperglycaemia in diabetes results in endothelial dysfunction and oxidative stress, contributing to frailty by reducing physical performance and impairing muscle function [46].

Furthermore, hyperglycaemia has been linked to cognitive decline, a significant component of frailty. Elevated blood glucose levels are associated with neurodegenerative changes that impair cognitive function over time. Cognitive impairment limits the ability to manage diabetes and other chronic conditions, leading to an accelerated decline into frailty. The presence of multiple comorbidities, such as cardiovascular disease and chronic kidney disease, further compounds the risk of frailty in individuals with chronic hyperglycaemia. These comorbid conditions place additional strain on the body’s physiological systems, reducing overall functional reserves and making it more difficult for individuals to recover from illness or injury [44,45].

Hyperglycaemia can be classified into mild, moderate, and severe levels, each of which has varying implications for the elderly, particularly in relation to frailty.

Mild hyperglycaemia is typically defined as fasting plasma glucose levels between 7.0 and 8.3 mmol/L (126–150 mg/dL) or HbA1c levels between 48 and 53 mmol/mol (6.5–7.0%) [47]. At this stage, hyperglycaemia is often asymptomatic, but prolonged exposure to mildly elevated blood glucose levels can have insidious effects on the body and contribute to chronic low-grade inflammation, which over time can lead to muscle weakness and a gradual decline in physical resilience. Even at these relatively modest levels, chronic hyperglycaemia can increase the risk of developing frailty by contributing to the subtle yet cumulative decline in bodily function, driven by the slow degradation of physiological reserves, leading to a higher likelihood of frailty in the long term [48].

Moderate hyperglycaemia is characterised by fasting plasma glucose levels between 8.3 and 13.9 mmol/L (150–250 mg/dL) or HbA1c levels between 53 and 64 mmol/mol (7.0–8.0%) [47]. At these levels, hyperglycaemia promotes the accumulation of advanced glycation end products (AGEs), which are implicated in the acceleration of both sarcopenia (muscle wasting) and cognitive decline, two critical components of frailty. AGEs are known to damage cellular structures and contribute to oxidative stress, further impairing physical and cognitive functions in older adults. Moreover, moderate hyperglycaemia has been linked to reductions in functional capacity, including mobility and balance, which can increase the risk of falls and further deterioration in frail older adults [45,46,49]. 

Severe hyperglycaemia is defined as fasting plasma glucose levels above 13.9 mmol/L (250 mg/dL) or HbA1c levels above 64 mmol/mol (8.0%) [47]. Severe hyperglycaemia is often associated with acute complications such as dehydration, electrolyte imbalances, and hyperosmolar hyperglycaemic state (HHS), which significantly impair physical function and cognition [49,50]. These complications can lead to an increased risk of hospitalisation and long-term disability, further exacerbating frailty in elderly individuals [28]. Severe hyperglycaemia has also been shown to accelerate cardiovascular and neurological complications, both of which contribute to the progression of frailty [44,45].

The presence of comorbidities, including cardiovascular disease, neuropathy, and renal dysfunction, further elevates the risk of frailty in individuals with diabetes [51]. Research has consistently demonstrated that individuals with diabetes have a higher prevalence of frailty than those without. According to a meta-analysis, diabetes was linked to a significantly elevated risk of frailty [52]. The relationship between diabetes and frailty is bidirectional. While diabetes can induce frailty, frailty can complicate diabetes management and worsen clinical outcomes. Frail individuals often exhibit diminished functional capacity, poor nutritional status, and reduced physical activity levels, all of which exacerbate diabetes control issues. Frailty can negatively impact adherence to diabetes medications and self-management practices, leading to suboptimal glycaemic control and an elevated risk of complications [46]. Prognosis and clinical outcomes in the elderly are significantly influenced by the interplay between diabetes and frailty. Frailty has been shown to be a substantial predictor of mortality and hospitalisation in elderly adults with diabetes [53]. This highlights the importance of identifying and addressing frailty in diabetes management to improve outcomes.

Comprehensive management strategies are necessary due to the intricate relationship between diabetes and frailty. Integrating geriatric and diabetes care is imperative to effectively address the multifaceted requirements of this population. Comprehensive Geriatric Assessment (CGA) can be crucial in identifying frailty in older individuals with diabetes and developing personalised care plans by assessing medical, functional, cognitive, and social domains [5]. This approach ensures that interventions are tailored to the patient’s unique needs and capabilities, enhancing the overall management of diabetes and frailty as illustrated in Figure 2.

Pharmacological management of frailty requires careful consideration. Older adults who are frail and have diabetes may be more susceptible to the adverse effects of diabetes medications, such as hypoglycaemia [54]. Consequently, treatment regimens should prioritise safety and simplicity, avoiding polypharmacy and reducing the risk of hypoglycaemia. Modifying glycaemic targets to reflect the individual’s level of frailty can prevent overtreatment and its associated risks.

## 5. Diabetes, Pharmacology, and Frailty

Given the complexity of controlling diabetes in the frail elderly population and the increased susceptibility of frail individuals to adverse drug reactions, a significant emphasis should be placed on the review of diabetic medications. Frequent medication reviews can facilitate the optimisation of treatment plans, reduce polypharmacy, lead to a reduction in risks, and result in improved outcomes [54,55]. The goals of managing diabetes in frailty should move from strict glucose control towards simplicity, preventing hypoglycaemia and improving quality of life. Strict glycaemic control is beneficial in younger populations, but it can lead to severe hypoglycaemia in the elderly, which is linked to a higher risk of morbidity and mortality [46]. Elderly patients with lower physiological reserves are more likely to be impacted by the negative consequences of hypoglycaemic episodes. Medication reviews can help to balance the risks and benefits of treating diabetes and can facilitate the de-escalation of aggressive therapies in favour of safer, more controllable regimens. Diabetes treatment plans should consider comorbidities, functional status, and cognitive abilities and should be adjusted to ensure that they are appropriate for the patient.

To achieve strict glycaemic control, polypharmacy with multiple antihyperglycaemic drugs is frequently used in the management of diabetes. However, strict glycaemic targets can increase the risk of hypoglycaemia, especially in frail older adults. Hypoglycaemia has serious consequences, including falls, cardiovascular events, and increased mortality. Reassessing glycaemic targets to weigh up the benefits and risks of hyperglycaemia and hypoglycaemia can help facilitate deprescribing. This may involve reducing or discontinuing medications such as sulfonylureas or certain insulin regimens that are associated with a higher risk of hypoglycaemia [54]. Evidence supports the use of less intensive glycaemic targets and simplified medication regimens to reduce the incidence of hypoglycaemia while maintaining adequate glycaemic control for this population, thus improving patient safety and quality of life [51]. 

Antihyperglycaemic agents including insulin, metformin, sulfonylureas, GLP-1 agonists, and SGLT2 inhibitors are useful in managing blood glucose levels of diabetic patients but their use in the elderly population can exacerbate frailty and contribute to its associated complications Table 3. The mechanisms by which these agents contribute to frailty include hypoglycaemia, loss of muscle mass, dehydration, and cognitive impairment [56].

The use of insulin is necessary for many diabetic patients, but it poses significant risks, particularly for frail older adults. One of the most critical concerns is the increased risk of hypoglycaemia. Hypoglycaemic episodes can result in severe consequences, such as confusion, dizziness, falls, fractures, and hospitalisations, which can directly exacerbate frailty by accelerating the decline in physical function and increasing vulnerability to further health complications [71,72]. Recurrent episodes of hypoglycaemia also contribute to cognitive impairment and dementia, further exacerbating frailty in older adults [73].

Metformin is widely used as a first-line treatment for type 2 diabetes due to its efficacy and generally low risk of hypoglycaemia. However, in frail older adults, metformin can contribute to frailty through gastrointestinal side effects such as nausea, vomiting, and diarrhoea, leading to dehydration and malnutrition, particularly in those with reduced physiological reserves. In elderly individuals and those with chronic kidney disease (CKD), metformin carries a risk of lactic acidosis, which can worsen frailty and lead to further complications [74]. Metformin may need to be stopped in elderly patients with poor renal function and is contraindicated in patients with a glomerular filtration rate (GFR) of less than 30 mL/min due to the risk of lactic acidosis [20]. An alternative medication is linagliptin, which is safer in those with reduced renal function.

Sulfonylureas, such as gliclazide, glimepiride, and glyburide, are another class of antihyperglycaemic agents associated with a high risk of hypoglycaemia, especially in the elderly. As with insulin, hypoglycaemic episodes caused by sulfonylureas can lead to physical injury, cognitive impairment, and increased frailty [56]. This risk is heightened in older adults with renal insufficiency, as the kidneys play a critical role in metabolising sulfonylureas [75]. Therefore, frail elderly patients taking sulfonylureas face an increased likelihood of severe hypoglycaemia, which can precipitate frailty-related complications such as falls and fractures. Since sulfonylureas and other oral antidiabetic drugs carry a significant risk of hypoglycaemia, they may not be appropriate for elderly patients who are cognitively impaired or have irregular eating habits [51,76].

GLP-1 receptor agonists, such as liraglutide, have shown benefits in terms of weight loss and glycaemic control, with a relatively low risk of hypoglycaemia. However, in frail older adults, weight loss induced by GLP-1 agonists can be detrimental in the elderly, leading to sarcopenia, which is characterised by the loss of muscle mass and strength [77]. Sarcopenia further exacerbates frailty by impairing mobility and balance and increasing the risk of falls. In frail individuals with already limited muscle reserves, this unintended weight loss can accelerate the decline in physical function and recovery from illness or injury [78].

SGLT2 inhibitors, such as empagliflozin and canagliflozin, are effective at lowering blood glucose levels and provide cardiovascular and renal benefits. However, they are associated with an increased risk of dehydration and electrolyte imbalances, particularly in older adults. SGLT2 inhibitors promote glucose excretion through urine, leading to diuresis, which can result in dehydration and orthostatic hypotension in frail elderly patients. Dehydration exacerbates physical frailty by reducing blood volume and impairing cardiovascular function, which can increase the risk of syncope, falls, and further decline in cognitive and physical function. In addition, the use of SGLT2 inhibitors may increase the risk of urinary tract infections, which can further complicate frailty in elderly patients [79,80], Table 3.

Poor adherence to complicated drug regimens can occur in the elderly with cognitive decline, reduced physical dexterity, and lack of social support [31]. Regular medication reviews help with patient education and adherence to treatment. Exercise and physical activity are essential parts of managing diabetes and frailty. Regular physical activity can improve muscle strength, physical performance, and glycaemic control, reducing the impact of both conditions [81]. Resistance and aerobic exercise programmes have been shown to be especially effective in improving functional outcomes and lowering the risk of frailty in older adults with diabetes.

Nutritional advice and guiding patients on adequate protein intake, along with micronutrients like vitamin D and antioxidants, can promote muscle health and metabolic function. Individualised dietary plans that address both glycaemic control and nutritional needs can help prevent and manage frailty in people with diabetes [82].

Regular assessments of diabetic medications are crucial for managing frailty and minimising negative consequences. Medication reviews prevent polypharmacy, reduce hypoglycaemia risk, drug–drug interactions, and improve adherence, all of which contribute to the best possible care for frail older persons with diabetes. Those with physical or cognitive impairments may find it difficult to follow complicated insulin regimens that call for regular monitoring and adjustment. By ensuring that treatment plans are safe and efficient, this holistic approach helps improve the quality of life and outcomes for this vulnerable population. Setting appropriate glycaemic targets in this population is also essential. In frailty, an HbA1c target of 7.5% is often recommended to balance the risks of strict glycaemic control with the potential benefits [20].

## 6. Hypertension and Frailty

Hypertension and frailty are closely linked conditions and appreciating the relationship between them is important in formulating effective management strategies to enhance outcomes in this population [57,83]. Hypertension is a well-established risk factor for the development of frailty. Chronically elevated blood pressure can lead to vascular damage and impaired blood flow, causing end-organ damage, reducing physical and cognitive function, and contributing to frailty [57,58]. Comorbidities like cardiovascular disease, renal dysfunction, and cerebrovascular disease, which are prevalent in this population, further compound the risk of developing frailty [84,85]. Similarly to diabetes and frailty, there is a bidirectional relationship between hypertension and frailty. Individuals who are frail often have reduced functional capacity, nutrition, and physical activity, all of which can also contribute to elevated blood pressure. Frailty can also impact medication adherence and self-management, especially where complex medication regimens are used, resulting in suboptimal blood pressure control and increased risk of complications [20,83]. Comprehensive geriatric assessment plays an important role in identifying frail individuals who have hypertension and then implementing personalised care plans by assessing medical, functional, cognitive, and social domains [5] (Figure 2).

## 7. Hypertension, Pharmacology, and Frailty

The management of hypertension in frail individuals requires a careful balance between achieving adequate blood pressure control and minimising the risk of adverse effects. Standard antihypertensive therapies may need to be adjusted to reduce the risk of side effects like orthostatic hypotension, which can lead to falls, fractures, and other complications [57,86]. Elderly and frail individuals have an increased sensitivity to antihypertensive medications, making them more vulnerable to the consequences of adverse drug reactions [5]. Blood pressure targets in older adults have been debated. Guidelines from the National Institute for Health and Care Excellence (NICE) recommend a target systolic blood pressure of <150 mmHg in the severely frail elderly population, highlighting the importance of individualised treatment plans [87]. Studies like the Systolic Blood Pressure Intervention Trial (SPRINT) have shown the benefits of intensive blood pressure control but often exclude frail individuals [88]. Bogaerts et al. [22] provided a systematic overview of the targets for blood pressure management in ageing and/or frailty, concluding that blood pressure targets for older people were <150 mmHg.

Regular medication reviews can help to identify and facilitate discontinuation of unnecessary medications, adjustment of doses, and switching to alternative antihypertensives. Studies have shown that individualised blood pressure targets in frailty can be beneficial Table 3. Williamson et al. found that intensive blood pressure control in older adults did not significantly reduce the risk of cardiovascular events or mortality when compared to less aggressive blood pressure targets [58]. Non-pharmacological interventions are also important to consider and can be very effective in managing hypertension. Dietary changes, increased physical activity, and reduction in weight in individuals with higher body mass index (BMI) can all lead to improved blood pressure control [84]. Addressing social aspects of health such as access to healthcare and social support is also important in the holistic management of frailty and hypertension. Exercise programs, including both aerobic and resistance training, can help reduce blood pressure and improve physical function in frail older adults [57]. These interventions not only assist in managing hypertension but also help to combat the physical decline associated with frailty. Cognitive and social support interventions can also benefit frail older adults with hypertension by addressing the psychosocial factors that contribute to both conditions [83,88].

## 8. The Role of Geriatricians and Pharmacists in Hospitals and the Community in Contributing to Deprescribing in Frailty, Diabetes, and Hypertension

Deprescribing, or the process of gradually discontinuing medications that are no longer beneficial or may cause harm, is an important strategy for reducing these risks and improving the health and quality of life of older adults [85]. The hazards associated with polypharmacy are especially relevant to frail patients because of their altered pharmacokinetic and pharmacodynamic profiles. For instance, decreased renal function, which is common in frail older adults, can cause some medications to accumulate, raising the possibility of toxicity [89]. Healthcare professionals can simplify treatment plans and lessen pharmacological burdens by conducting routine medication reviews, which help to identify unnecessary or potentially hazardous prescriptions. It takes a comprehensive approach to patient care, including regular medication reviews, patient and carer education, and collaborative decision-making. By involving patients in the deprescribing process, healthcare providers can ensure that the treatment is consistent with the patient’s values and preferences, resulting in improved adherence and satisfaction with care. For example, discontinuing medications that cause sedation, orthostatic hypotension, or cognitive impairment can significantly reduce the risk of falls and improve cognitive function [55]. Targeted deprescribing interventions have been shown in studies to improve outcomes such as medication burden, adverse drug events, and functional status [85].

**Figure 3:**
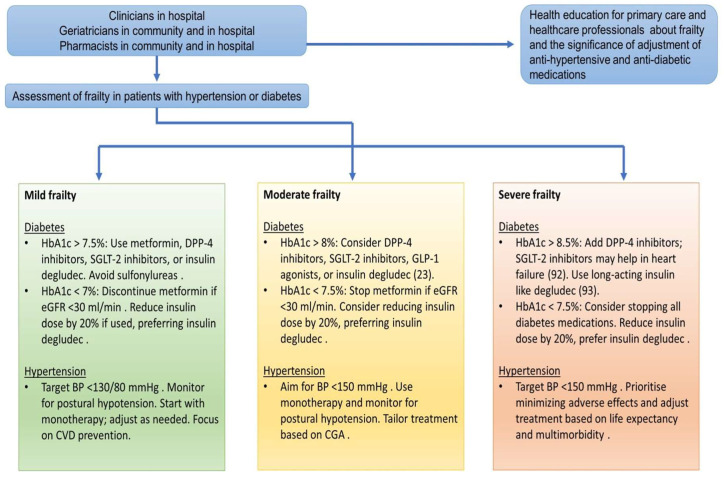
The role of geriatricians, clinicians, and pharmacists in hospitals and the community in contributing to deprescribing in frailty, diabetes, and hypertension. The role of geriatricians, clinicians, and pharmacists is expected to involve health education for health professionals in the community.

Polypharmacy poses significant risks in the management of frailty, diabetes, and hypertension in older adults. Deprescribing is an important strategy for reducing these risks, improving patient safety, and increasing quality of life. Healthcare providers can effectively manage these complex conditions while minimising the negative effects of polypharmacy by carefully evaluating medication regimens, setting individualised treatment goals, and adopting a patient-centred approach. The first step is to identify and assess frailty and then perform a Comprehensive Geriatric Assessment. The degree of frailty can then be used to determine the best strategies for deprescribing antidiabetic and antihypertensive medications. Table 4 explains suggested strategies for deprescribing in fit and well/prefrailty, moderate frailty, and severe frailty in individuals with diabetes and hypertension. Determination of frailty status should be part of the routine practice by clinicians, community and hospital geriatricians, and hospital and community pharmacists in all old populations with diabetes and hypertension. It is expected that all of these health professionals should also be engaged in health education of primary care health professionals about frailty, diabetes, and hypertension. Determination of frailty levels, blood pressure reading and monitoring, blood glucose, and HbA1c will help health professionals in hospitals or communities to decide about deprescribing or intensifying medication therapy (Figure 3). Health education for health professionals in the community is vital and should be conducted by community and hospital geriatricians and pharmacists and input from clinicians like diabetologists and nephrologists should be encouraged (Figure 3).

## 9. Future Directions

While current research highlights the importance of frailty assessment and deprescribing in the management of chronic conditions among the elderly, significant gaps remain that future research must address (Table 5). Investigating novel frailty biomarkers and developing more sensitive and specific frailty assessment tools could allow for earlier identification and tailored interventions in patients with hypertension and diabetes. Additionally, exploring the long-term impact of deprescribing on frailty progression and overall survival will provide clearer insights into how antihypertensive and diabetes medication optimisation can best serve this population. Future research should also focus on refining antihypertensive and diabetes medications and non-pharmacological interventions that consider the unique needs of frail individuals with multiple chronic conditions, particularly those commonly excluded from clinical trials.

By testing our hypothesis (deprescribing of diabetes and antihypertensive medications in the frail elderly population) through longitudinal population studies, we can further understand how these interventions impact frailty progression and overall health. Large-scale, randomised controlled trials that include frail and multimorbid patients could better inform evidence-based guidelines for the elderly. These studies should be carefully designed and monitored to avoid inducing harm and to ensure adherence to the ethical code of medical practice. Furthermore, understanding patient and caregiver perspectives on deprescribing could help develop more patient-centred approaches, ensuring that deprescribing strategies align with the preferences and goals of elderly individuals. Overall, the integration of personalised medicine and frailty management in people with diabetes and hypertension holds the potential to improve the quality of life and outcomes for the growing elderly population. It is of great importance to assess the impact of deprescribing on parameters such as hospital admissions, falls, and, most importantly, fractures of the neck of the femur (NOF). Orthogeriatricians frequently deal with patients presenting with NOF as a result of falls, especially those with diabetes and hypertension. Therefore, it is important to emphasise that such clinical trials can be conducted by orthogeriatricians in their wards to assess mortality and morbidity associated with deprescribing in frail elderly populations.

## 10. Limitations and Strengths

This narrative review involved an extensive literature search on the topic, which may have led to bias in the selection of studies and further interpretation, as there is potential variation in the studies included. This contrasts with a systematic review, where the focus is on the quality of the studies selected and included. Another limitation is that we included only studies published in the English language. Strengths of our review include the use of a variety of studies from different countries, allowing a richer, more nuanced perspective on the topic. This review sets the stage for the current research evidence available on the importance of deprescribing in the frail elderly population with diabetes and hypertension. This extensive literature search provides an excellent projection for the future direction of research in this field by identifying gaps in the literature. Importantly, this review is applicable to a wide variety of clinicians, including geriatricians in hospitals and the community, orthogeriatricians, acute medicine physicians, family medicine and primary care physicians, as well as hospital and community pharmacists. Healthcare professionals dealing with frail elderly populations in hospitals or the community will find this review relevant and responsive to their educational needs.

## 11. Conclusions

In this review, we highlight the critical interplay between frailty, diabetes, hypertension, polypharmacy, and deprescribing in the elderly and frail population. We also emphasise the critical role of geriatricians and pharmacists in hospitals and communities in the management of antihypertensive and diabetes medications. We found that diabetes and hypertension, which are common among the elderly, play a significant role in the progression of frailty. These conditions frequently require complex medication regimens, resulting in polypharmacy, which carries additional risks such as adverse drug reactions, increased hospitalisations, and further morbidity. Polypharmacy is a common issue in elderly patients with multiple comorbidities and was identified as a major contributor to frailty. Importantly, deprescribing in frail elderly patients with diabetes and hypertension lowers the risk of adverse effects such as orthostatic hypotension, hypoglycaemia, and drug interactions. By lowering these risks, deprescribing can help prevent falls, reduce hospitalisations, and improve functional status and medication adherence. Deprescribing should be approached from a patient-centred perspective, ensuring that it aligns with the patient’s health goals and preferences. Prioritising deprescribing as a key component of frailty management allows healthcare providers to significantly contribute to the well-being and longevity of elderly patients. Future large population studies are needed to assess the best approaches for managing diabetes and hypertension in frail individuals.

## Figures and Tables

**Table 1:** Summary of the components of the Clinical Frailty Scale. For the full details of the components of the scale, please see Reference [30].

Clinical Frailty Scale	
1—Very fit	Among the fittest for their age (robust, active, energetic, motivated, and regularly exercise).
2—Well	No severe disease symptoms but is less fit than category 1 and exercise or are very active occasionally.
3—Managing	Medical problems are well controlled but not regularly active beyond routine walking.
4—Very mild frailty	While not dependent on others for daily help, symptoms often limit activities (decline in activity and tiredness).
5—Mild frailty	More evident slowing and need help in higher-order instrumental activities of daily living (IADLs) such as finance, transportation, heavy housework, and medication management. Need support with outdoor activities.
6—Moderate frailty	Need help with all outside activities, housekeeping, and daily living.
7—Severe frailty	Completely dependent for cognitive and physical personal care (stable and not at high risk of dying).
8—Very severe frailty	Completely dependent for personal care and approaching the end of life.
9—Terminally ill	Approaching the end of life.

**Table 2:** Summary of the components of the Edmonton Frail Scale. For more details on the assessment of all of these domains please see Reference [33].

Edmonton Frail Scale				
Frailty Domain	Summary Of Assessment of The Domain	0 Point	1 Point	2 Points
Cognition	Clock test	No errors	Minor spacing errors	Other errors
General health status	Frequency of hospital admission during the last year	0	1–2	≥2
	In general, how would you describe your health?	Excellent, Very good, Good	Fair	Poor
Functional independence	Help with outdoor and indoor activities	0–1	2–4	5–8
Social support	Someone who is willing and able to meet the need	Always	Sometimes	Never
Medication use	Using five medications on a regular basis	No	Yes	
	Do you forget to take your medication?	No	Yes	
Nutrition	Have you recently lost weight?	No	Yes	
Mood	Do you often feel sad or depressed?	No	Yes	
Continence	Do you have a problem with losing control of urine?	No	Yes	
Functional performance	Sitting, stand up, and walk task for 3 m and return	0–10 s	11–20 s	>20 s Unwilling, needs assistance
Total points out of 17				

Scoring: 0–5 = not frail; 6–7 = vulnerable; 8–9 = mild frailty; 10–11 = moderate frailty; and 12–17 = severe frailty.

**Table 3:** Summary of antidiabetic and antihypertensive medications with their benefits and risks [20,54,57,58].

Medication Class	Examples	Benefits	Risks
Biguanides	Metformin	Widely used, improves CVD [23]	Lactic acidosis, may increase weight loss, and B12 deficiency [23]
DPP-4 inhibitors	Linagliptin	Can be used in CKD [23]	Low risk of hypoglycaemia [23]
Sulfonylureas	Glimepiride	Cost-effective, widely used and can be used with other diabetes medications [23]	High risk of hypoglycaemia in frail patients [23]
GLP-1 agonists	Liraglutide, exenatide	Weight loss, cardiovascular benefits [23]	Risk of pancreatitis, weight loss, frailty, sarcopenia, and needs to be injected [23]
SGLT2 inhibitors	Empagliflozin, canagliflozin	Cardiovascular and renal benefits [23]	Risk of dehydration, urine infection, weight loss, and euglycaemic ketoacidosis [23]
Glitazones	Pioglitazone, rosiglitazone	Well tolerated [23]	Not widely used (heart failure, osteoporosis, and bladder cancer) [23]
Short-acting insulin	Actrapid, Humulin S	Flexible dosing [23]	Risk of hypoglycaemia, monitoring of glucose, and assistance with injection [23]
Intermediate insulin	Isophane insulin	Well tolerated, cheap [23]	Risk of hypoglycaemia, twice injection per day, and may lead to weight gain [23]
Long-acting basal insulin	Insulin glargine, detemir	One injection a day, less risk of hypoglycaemia in comparison with isophane insulin [23]	Risk of hypoglycaemia [23]
Ultra-long-acting insulin	Insulin degludec, insulin glargine U300	Risk of hypoglycaemia is less than long-acting insulin [59]	Expensive insulin and cost may decrease in the future [59]
Thiazide diuretics	Hydrochlorothiazide	Effective, reduces fluid retention [60]	Risk of electrolyte imbalance [61]
Calcium channel blockers	Amlodipine, diltiazem	Effective, good side effect profile [62]	Risk of oedema and dizziness [62]
Beta-blockers	Metoprolol, atenolol	Effective in heart disease [63]	Risk of bradycardia and fatigue [63]
ACE inhibitors	Lisinopril, enalapril	Kidney protective [64]	Risk of hyperkalaemia and cough [65]
Angiotensin receptor blockers	Losartan, valsartan	Kidney protective [66]	Risk of hyperkalaemia and dizziness [66]
α-adrenoreceptor antagonists (α-blockers)	Prazosin, doxazosin	Effective for hypertension, can be used for BPH [67]	Risk of postural hypotension and dizziness [67]
Aldosterone antagonists	Spironolactone, eplerenone	Effective for heart failure, reduces fluid retention [68]	Risk of hyperkalaemia; risk of gynecomastia with spironolactone [69]
Central α-adrenoreceptor agonists	Clonidine, methyldopa	Effective for resistant hypertension [70]	Risk of sedation, dry mouth, and rebound hypertension [70]

**Table 4:** Summary of management of hypertension and diabetes in the elderly with mild, moderate, and severe frailty. The main references are included.

	Prefrail/Fit and Well	Moderate Frailty	Severe Frailty
**Diabetes**	**HbA1c > 7.5%:** Use metformin, DPP-4 inhibitors, SGLT-2 inhibitors, or insulin degludec. Avoid sulfonylureas [23]. **HbA1c < 7%:** Discontinue metformin if eGFR < 30 mL/min. Reduce insulin dose by 20% if used, preferring insulin degludec [90].	**HbA1c > 8%:** Consider DPP-4 inhibitors, SGLT-2 inhibitors, GLP-1 agonists, or insulin degludec [23]. **HbA1c < 7.5%:** Stop metformin if eGFR < 30 mL/min. Consider reducing insulin dose by 20%, preferring insulin degludec [91].	**HbA1c > 8.5%:** Add DPP-4 inhibitors; SGLT- 2 inhibitors may help in heart failure [92]. Use long-acting insulin like degludec [93]. **HbA1c < 7.5%:** Consider stopping all diabetes medications. Reduce insulin dose by 20%, prefer insulin degludec [91].
**Hypertension**	Target BP < 130/80 mmHg [58]. Monitor for postural hypotension. Start with monotherapy; adjust as needed. Focus on CVD prevention [57,94].	Aim for BP < 150 mmHg [58]. Use monotherapy and monitor for postural hypotension. Tailor treatment based on CGA [57].	Target BP < 150 mmHg [58]. Prioritise minimising adverse effects and adjust treatment based on life expectancy and multimorbidity [57].

**Table 5:** Summary of main systematic reviews, meta-analyses, and randomised clinical trials included in this paper that discuss the management of diabetes and hypertension in the elderly frail population.

Role of Frailty in:	Summary of Findings	References
**Diabetes Management**	Diabetes increases the risk of frailty through inflammatory and vascular pathways. Frailty complicates diabetes management and leads to higher complication rates and suboptimal glycaemic control. Regular diabetes medication reviews decrease the risk of hypoglycaemia and improve patient outcomes.	[13,20,46,51,52,95]
**Hypertension Management**	Individualised blood pressure targets minimise adverse effects in frail patients. Less aggressive blood pressure targets are recommended for severely frail individuals. Diet and exercise are crucial for managing hypertension and improving physical function in frail older adults.	[22,57,58,96,97,98,99]
**Nutritional Interventions**	Malnutrition in frail older adults necessitates nutritional interventions to improve outcomes. Regular physical activity and personalised dietary plans promote muscle health and metabolic function. Exercise programs including resistance and aerobic training help reduce frailty and improve functional outcomes.	[3,43,81]
**Deprescribing and Polypharmacy**	Deprescribing reduces adverse drug events and improves functional status. Medication reviews are useful in excluding unnecessary medications, improving treatment plans, and reducing polypharmacy. Effective deprescribing requires a multidisciplinary approach involving regular medication reviews and patient education.	[84,85]
**Frailty Assessment**	Tools like the Fried Frailty Phenotype and Clinical Frailty Scale are validated for assessing frailty. CGA improves outcomes such as survival and the likelihood of staying at home post-discharge.	[6,30,39]

## Data Availability

Not applicable.
